# The Impact of UFP-512 in Mice with Osteoarthritis Pain: The Role of Hydrogen Sulfide

**DOI:** 10.3390/antiox12122085

**Published:** 2023-12-07

**Authors:** Gerard Batallé, Xue Bai, Gianfranco Balboni, Olga Pol

**Affiliations:** 1 Grup de Neurofarmacologia Molecular, Institut de Recerca Sant Pau, 08041 Barcelona, Spain; 2 Grup de Neurofarmacologia Molecular, Institut de Neurociències, Universitat Autònoma de Barcelona, 08193 Barcelona, Spain; 3 Unit of Pharmaceutical, Pharmacological and Nutraceutical Sciences, Department of Life and Environmental Sciences, University of Cagliari, 09042 Cagliari, Italy

**Keywords:** analgesia, antioxidants, anxiety, δ-opioid receptors, depression, osteoarthritis pain, oxidative stress

## Abstract

The pain-relieving properties of opioids in inflammatory and neuropathic pain are heightened by hydrogen sulfide (H_2_S). However, whether allodynia and functional and/or emotional impairments related to osteoarthritis (OA) could be reduced by activating δ-opioid receptors (DOR) and the plausible influence of H_2_S on these actions has not been completely established. In female C57BL/6J mice with OA pain generated via monosodium acetate (MIA), we analyze: (i) the effects of UFP-512 (a DOR agonist), given alone and co-administered with two H_2_S donors, on the symptoms of allodynia, loss of grip strength (GS), and anxiodepressive-like comportment; (ii) the reversion of UFP-512 actions with naltrindole (a DOR antagonist), and (iii) the impact of UFP-512 on the expression of phosphorylated NF-kB inhibitor alpha (p-IKBα) and the antioxidant enzymes superoxide dismutase 1 (SOD-1) and glutathione sulfur transferase M1 (GSTM1); and the effects of H_2_S on DOR levels in the dorsal root ganglia (DRG), amygdala (AMG), and hippocampus (HIP) of MIA-injected animals. Results showed that systemic and local administration of UFP-512 dose-dependently diminished the allodynia and loss of GS caused by MIA, whose effects were potentiated by H_2_S and reversed by naltrindole. UFP-512 also inhibited anxiodepressive-like behaviors, normalized the overexpression of p-IKBα in DRG and HIP, and enhanced the expression of SOD-1 and GSTM1 in DRG, HIP, and/or AMG. Moreover, the increased expression of DOR triggered by H_2_S might support the improved analgesic actions of UFP-512 co-administered with H_2_S donors. This study proposes the use of DOR agonists, alone or combined with H_2_S donors, as a new treatment for OA pain.

## 1. Introduction

Osteoarthritis (OA) is a degenerative illness that causes chronic pain and which affects about 10–20% of the population over 50 years old [1,2]. OA is a prevalent disease in society, particularly among women (35%), and a cure remains unidentified [3]. The clinical manifestations of OA involve joint functional weaknesses, pain, impairment in walking, and other psychological symptoms, such as depression and anxiety, which can exacerbate pain [4]. Although there are several therapies for OA pain, such as acetaminophen, nonsteroidal anti-inflammatory compounds, and opioids, they do not provide enough pain relief, and their repetitive use is often associated with major side effects [5]. Thus, the search for treatments capable of resolving the functional and affective disorders accompanying OA pain with low side effects is required.

The opioid system plays a pivotal function in pain management. Previous findings have demonstrated the analgesic effects of µ-(MOR), δ-(DOR) and/or k-opioid receptor agonists [6,7,8,9] and their involvement in the control of affective disorders [10,11,12,13]. MOR are the main target of opioids, but morphine and other MOR agonists also have important side effects, including tolerance, constipation, nausea, vomiting and addiction [14]. Conversely, DOR agonists produce analgesia with fewer unwanted effects [7,15]. Some evidence also indicates that while DORs have only low analgesic properties in normal conditions [16], they play a critical role in regulating inflammatory pain [17] as confirmed by the increased inflammatory pain observed in DOR knockout mice [18]. DORs are fully distributed in the peripheral nervous system (PNS) and in several brain regions, including the amygdala (AMG), cortex, hippocampus (HIP) and periaqueductal gray matter [12,19]. In fact, DORs additionally modulated the emotional responses in several preclinical models of depression and anxiety [13] as well as in mice with depressive-like behaviors concurrent with neuropathic pain [20]. However, the effects of DOR agonists on pain relief and on the modulation of the functional and affective deficits related to OA pain have not been completely explored.

NF-B activation is important for the development of the inflammatory responses, which contribute to the death of chondrocytes and the destruction of cartilage [21]. Transcription factor NF-Κb, besides controlling the synthesis of pro-inflammatory cytokines, also modulates redox balance by regulating the expression of reactive oxygen species (ROS) [22]. Several studies have demonstrated the involvement of NF-κB in the progress of OA pain and various treatments, such as scutellarin, *Agrimonia pilosa* Ledeb and *Salvia miltiorrhiza* Bunge, inhibited OA pain by blocking its expression [23,24]. Nevertheless, the role played by this inflammatory factor in the analgesic actions of DOR agonists has not been completely assessed.

The gaseous neurotransmitter hydrogen sulfite (H_2_S) is considered beneficial due to its antioxidant, anti-inflammatory and neuroprotective properties [25]. Therefore, the systemic administration of slow-releasing H_2_S donors exerted antinociceptive action under diabetic neuropathy, inflammatory and OA pain conditions [25,26,27]. Current research has also demonstrated that the administration of H_2_S donors enhanced the effectiveness of DOR agonists in reducing allodynia and hyperalgesia caused by peripheral inflammation and nerve damage [28,29]. These findings reveal that the administration of DOR agonists in combination with H_2_S donors could be a promising strategy for the treatment of chronic pain. These experiments further demonstrated that the improved analgesic actions of DOR agonists induced by H_2_S were mainly produced by increasing the expression of DORs in the PNS and/or central nervous system (CNS) of rodents with inflammatory or neuropathic pain, thus showing a positive relationship between H_2_S and DORs in both pain models. Nonetheless, the potential impact of H_2_S on the antiallodynic and the recovery of grip strength (GS) induced by UFP-512 (a DOR agonist) during OA pain has not been assessed.

Various reports have revealed that oxidative stress contributes to central and peripheral sensitization and that Nrf2 plays a protective role in diminishing oxidative stress by activating many defensive enzymes, as it comprises superoxide dismutase 1 (SOD-1) and glutathione sulfur transferase M1 (GSTM1) [30,31]. Additionally, other studies have revealed that UFP-512 protects cells and inhibits inflammatory pain by activating the endogenous antioxidant system in the spinal cord of animals with peripheral inflammation [20,32]. Then, we investigated whether the activation of the antioxidant path could be implicated in the anti-allodynic, anxiolytic and/or antidepressant actions of UFP-512 in animals with OA pain.

We used a pain model of OA generated via intra-articular injection of monosodium acetate (MIA) in female mice to evaluate: (1) the effects of systemic and intra-articular administration of UFP-512, both alone and combined with two H_2_S donors, diallyl disulfide (DADS) and morpholin-4-ium 4-methoxyphenyl(morpholino) phosphinodithioate dichloromethane complex (GYY4137), on the allodynia and the decreased GS provoked by OA; (2) the anxiolytic and/or antidepressive actions of UFP-512; (3) the reversion of the effects produced by UFP-512 with the DOR antagonist, naltrindole; (4) the impact of UFP-512 on the levels of p-IKBα and the enzymes SOD-1 and GSTM1 as well as the effects produced by DADS and GYY4137 on the expression of DORs in the dorsal root ganglia (DRG), AMG and HIP of MIA-injected mice.

## 2. Materials and Method

### 2.1. Animals

Female C57BL/6 mice of 6–8 weeks old were obtained from Envigo Laboratories located in Barcelona, Spain. They were kept in a light/dark (12/12 h) conditions, with a temperature of 22 °C and a humidity of (55 ± 10%). They had free access to food and water. After a period of seven days of acclimatization to the environment, experiments were conducted between the hours of 9:00 a.m. and 5:00 p.m., in agreement with the guidelines of the European Commission directive (2010/63/EC) and the Spanish Law (RD 53/2013) regulating animal research and were approved by the local Committee of Animal Use and Care of the Autonomous University of Barcelona (ethical code: 4581). Every effort was made to reduce the number of animals used and the suffering of the animals. All experiments were performed under blinded conditions.

### 2.2. Materials

MIA, naltrindole, DADS, GYY4137, RIPA buffer, phosphate-buffered saline (PBS), serum albumin (BSA) and the primary antibody anti-glyceraldehyde-3-phosphate dehydrogenase (GAPDH) were purchased from Sigma-Aldrich (St. Louis, MO, USA). The primary antibodies anti-p-IKBα or DORs were obtained from Abcam (Cambridge, UK); the anti-IKBα from Cell Signaling Technology (Danvers, MA, USA) and the anti-SOD-1 or GSTM1 from Novus Biologic (Littleton, CO, USA). The secondary antibody horseradish peroxidase-conjugated anti-rabbit and the ECL kit were acquired from GE Healthcare (Little Chalfont, Buckinghamshire, UK).

UFP-512, synthesized by Balboni et al., (2002) [33] and naltrindole were dissolved in saline solution (NaCl 0.9%; VEH) and given intraperitoneally (i.p.) or intra-articularly (i.a.) at a volume of 10 mL/kg (i.p.) or 30 µL (i.a.), at 1 h or 30 min before testing. DADS and GYY4137 were also dissolved in VEH and i.p. injected at 10 mL/kg, 1 h before testing, according to other works [28,29]. All drugs were prepared immediately before use. Control animals received the corresponding volume of VEH.

### 2.3. The Induction of OA Pain

The mice right knee joints was shaved and flexed at a 90° angle and 10 µL of MIA (15 mg/mL) was i.a. injected into the joint space [34]. This process was carried out under brief anesthesia with isoflurane (induction 3% and maintenance 2.5%). MIA was dissolved in VEH. Control mice received the i.a. administration of VEH.

### 2.4. Allodynia and GS Measurements

The assessment of mechanical allodynia was conducted by evaluating the withdrawal response of the hind paw to stimulation of the von Frey filaments with varying bending forces (varying from 0.4 to 3 g). The animals were placed in Plexiglas cylinders (20 high × 9 diameter cm) on a grid bottom through which the filaments (North Coast Medical, Inc., San Jose, CA, USA) were applied utilizing the up–down paradigm [35]. The response threshold was estimated using an Excel program (Microsoft Iberia SRL, Barcelona, Spain). Prior to the experiment, mice were habituated to the environment for 1 h.

To measure the GS, the investigator held the mice by the base of the tail, permitting both hind paws to grip a metal bar connected to a force transducer (Model 47200; Ugo Basile; Varese, Italy) that automatically recorded the peak force for each measurement (g) [34]. The baseline GS values were recorded as the mean of three determinations before MIA or SS administration. The subsequent determinations were based on this value, which was considered 100% of GS.

### 2.5. Affective Behaviors

The evaluation of the anxiety-like behavior was carried out by employing the elevated plus maze (EPM) [36], a maze with four arms (5 cm wide/35 cm long) situated 45 cm above the floor. The animal was positioned in the center of the maze, looking into one open arm, and the sum of entries into open and closed arms and the percentage of time they stayed in the open arms, in the 5 min of the test, were evaluated. A low number of entrances and a short period of time spent in the open arms were considered anxious-like comportment.

The tail suspension test (TST) and the forced swimming test (FST) were employed to determine if depressive-like behaviors manifested in mice with OA pain [37,38]. In the TST, mice were suspended on a bar positioned 35 cm above the floor by means of adhesive tape located 1 cm from the tip of the tail. The immobility time (s) was recorded for 6 min. In the FST, mice were put into a transparent Plexiglas cylinder (25 cm/10 cm) filled with 10 cm of water (24 °C ± 0.1 °C). For each animal, behavior over 6 min was recorded, and the time that the subject remained immobile was assessed for the last 4 min. In these tests, increased immobility was considered a depressive-like behavior.

Before starting the tests, the animals were familiarized to the testing room for 1 h.

### 2.6. Western Blotting Analysis

The expression of p-IKB, SOD-1, GSTM1 and DORs in the DRG, AMG and HIP was evaluated at day 29 after MIA injection. Mice were euthanized via cervical dislocation, and tissues were dissected and preserved at −80 °C. The extracted tissues were sonicated in cold lysis buffer RIPA Buffer and solubilized (1 h at 4 °C). Homogenates were sonicated again for 10 s and then centrifuged (700× *g*, 20 min, 4 °C). Proteins were separated via electrophoresis with a 12% sodium dodecyl sulfate polyacrylamide gel and electrophoretically transferred onto a polyvinylidene fluoride membrane for 120 min. Membranes were blocked with PBS plus 5% nonfat dry milk, Tris-buffered saline with Tween 20 plus 5% BSA or 5% nonfat dry milk or with PBS with Tween 20 plus 5% BSA, for 75 min. Membranes were incubated with primary antibodies anti: p-IKBα (1:150), DOR (1:300), IKBα (1:150), SOD-1 (1:150), GSTM1 (1:150) or GAPDH (1:5000), overnight at 4 °C. After that, blots were incubated with a horseradish peroxidase-conjugated anti-rabbit secondary antibody for 1 h at room temperature, and proteins were detected using the chemiluminescence detection system. We used the Image-J program (National Institutes of Health, Bethesda, MD, USA) to do a densitometric analysis.

### 2.7. Experimental Protocol

Initially, we assessed the mechanical allodynia and GS deficits provoked by MIA at day 29 after its administration. In accordance with these results, the subsequent experiments were conducted at day 29 following MIA or VEH injection.

First, we analyzed the actions produced via the acute administration of different doses of UFP-512, 1–50 mg/kg or 250–1000 µg, i.p. or i.a. administered, on the mechanical allodynia and GS losses linked with OA and their reversion with the DOR antagonist naltrindole (3 mg/kg i.p. or 250 µg i.a.) (*n* = 6 animals per group). The doses of these drugs were chosen based on another research study [29].

The effects of a high dose of UFP-512 (50 mg/kg) and its reversion with naltrindole (3 mg/kg), both given i.p., on the anxiety- and depressive-related behaviors observed at 29 days following MIA injection were also evaluated (*n* = 8 animals per group).

To study the possible heightening induced via H_2_S on anti-allodynic actions and the recovery of GS produced via UFP-512, the outcomes of the systemic injection of low doses of GYY4137 (0.4 mg/kg) or DADS (3 mg/kg) combined with 3 mg/kg or 250 µg of UFP-512, i.p. or i.a. injected, at day 29 after MIA injection were analyzed (*n* = 6 animals per group).

Moreover, the effects of UFP-512 (50 mg/kg i.p.) on the protein levels of p-IKBα, SOD-1 and GSTM1 and those produced via DADS (15 mg/kg) and GYY4137 (6 mg/kg) on the expression of DORs in the DRG, AMG and HIP at 29 days following MIA injection were evaluated using Western blot assay (*n* = 3 samples per group). The doses of DADS and GYY4137 were based on a previous study which demonstrated their effectiveness in inhibiting OA pain [34]. VEH plus VEH-treated mice were used as controls.

### 2.8. Data Analyses

Data are given as means ± standard error of the mean (SEM). One-way ANOVA, followed by a Tukey post hoc test, was utilized for determining significant variations between groups in the behavioral and biochemical experiments. We used GraphPad Prism (version 9.0; La Jolla, CA, USA) for statistical analysis.

The anti-allodynic effects produced by treatments are depicted as the percentage of the maximal possible effect, which was determined by comparing the test latencies pre-(baseline) and post-drug injection and calculating them in accordance with this equation:

Maximal possible effect (%) = [(drug − baseline)/(cut-off—baseline)] × 100

The percentage of improvement in grip strength is showed by comparing the grip strength before (baseline) and after the drug injection. The equation used to calculate this effect is:

Recovery of grip strength (%) = [(drug − baseline)/(baseline)] × 100

A *p* value lower than 0.05 was considered significant.

## 3. Results

### 3.1. Treatment with UFP-512 Inhibits the Allodynia and GS Deficts Caused by MIA

**Figure 1:**
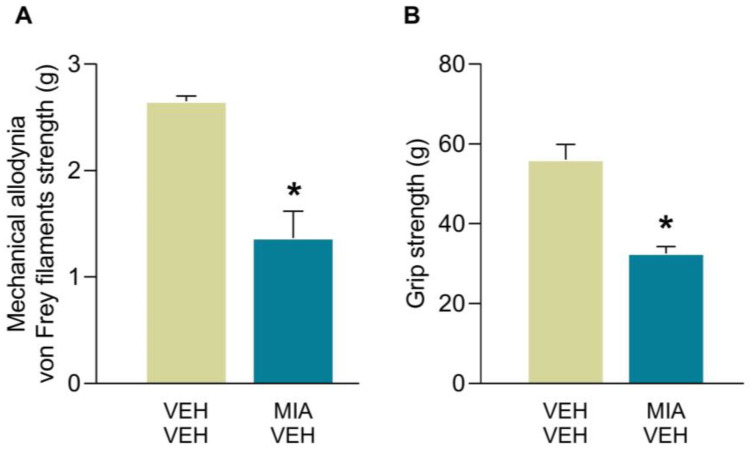
Mechanical allodynia and GS deficits caused by MIA in mice. The mechanical allodynia (von Frey filament strength, g) (**A**) and GS force deficits (g) (**B**) provoked by the intra-articular injection of MIA in the hind paws of animals at 29 days after its administration are represented. Results are showed as mean values ± SEM; *n* = 6 subjects per group. For each test, * *p* < 0.002 denotes significant differences between groups (unpaired Student’s *t* test).

In accordance with our previous findings, MIA given i.a. provoked mechanical allodynia and GS deficits at 29 days after its administration (Figure 1). Indeed, MIA injection led to a significant decrease on the threshold for evoking paw withdrawal to a mechanical stimulus and a reduction of the grip strength of the hind paws as compared to those of the animals treated with VEH (*p* < 0.0002; unpaired Student’s *t* test).

Then, in animals with mechanical allodynia and GS deficits triggered by MIA at 29 days after injection, we evaluated the effects produced by the intraperitoneal and intra-articular administration of UFP-512 and their reversion with naltrindole.

**Figure 2:**
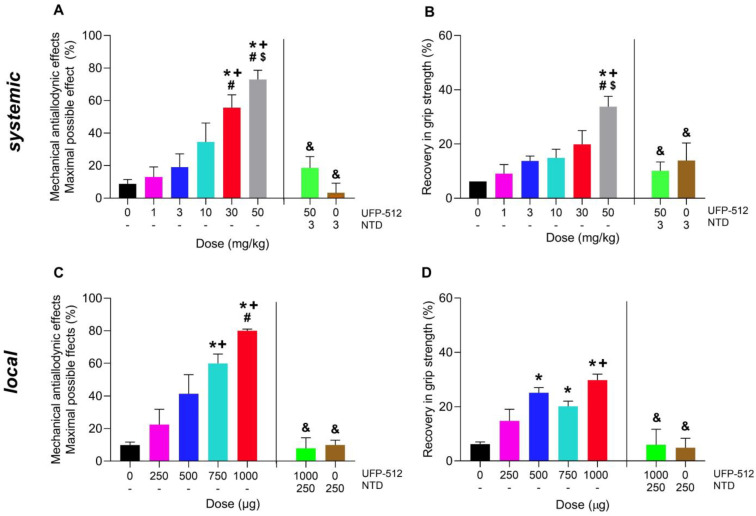
The reduction of the allodynia and GS deficits caused by OA produced by the systemic and local injection of UFP-512 and its reversion with naltrindole at 29 days followed MIA injection. The anti-allodynic effect (**A**,**C**) and recovery of GS (**B**,**D**) resulting from UFP-512 given i.p. (**A**,**B**) or i.a. (**C**,**D**) at 1 h or 30 min before testing are presented. The reversion of the inhibitory effects induced via 50 mg/kg or 1000 µg of UFP-512 with the systemic (3 mg/kg) or local (250 µg) administration of naltrindole (NTD) are also represented. For each test, symbols show significant differences; * vs. 0 mg/kg or 0 µg of UFP-512, + vs. 1 mg/kg or 250 µg of UFP-512; # vs. 3 mg/kg or 500 µg of UFP-512; $ vs. 30 mg/kg of UFP-512 and & vs. 50 mg/kg or 1000 µg of UFP-512 (*p* < 0.05; one-way ANOVA plus Tuckey test). The data are showed as mean ± SEM; *n* = 6 subjects.

Our results showed that treatment with UFP-512 administered i.p. (1–50 mg/kg) and i.a. (250–1000 µg) inhibited the mechanical allodynia and GS deficits caused by MIA in dose-dependent mode (Figure 2). Therefore, the anti-allodynic actions generated by 30 and 50 mg/kg of UFP-512 given i.p. in MIA-injected mice were higher than those resulting from low doses of this drug (0, 1, 3 and/or 10 mg/kg; *p* < 0.0001; one-way ANOVA; Figure 2A). Additionally, the recovery of GS resulting from 50 mg/kg of UFP-512 was greater than that of 0, 1, 3 and 10 mg/kg (*p* < 0.0001; one-way ANOVA; Figure 2B).

Regarding the effects produced by the local administration of UFP-512, our results also demonstrated that the anti-allodynic effects resulting from the i.a. administration of 750 and 1000 µg of UFP-512 were greater than those produced by 0, 250 and/or 500 µg (*p* < 0.0001; one-way ANOVA, Figure 2C), while the recovery of the loss of GS produced by 500, 750 or 1000 µg of UFP-512 were higher compared to those resulting from 0 and/or 250 µg of this DOR agonist (*p* < 0.0001; one-way ANOVA; Figure 2D).

Moreover, the i.p. (3 mg/kg) or i.a. (250 µg) administration of naltrindole, a selective DOR antagonist, reversed the anti-allodynic effects and the recovery of GS caused by high doses of UFP-512 given i.p. (50 mg/kg) or i.a. (1000 µg), respectively (Figure 2A–D).

### 3.2. The Impact of UFP-512 on the Affective Disorders Associated with OA Pain and Its Reversion with Naltrindole

**Figure 3:**
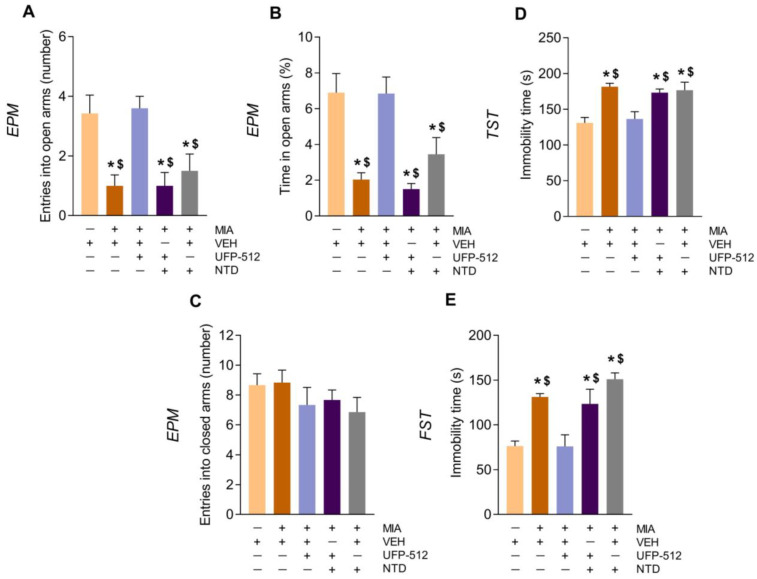
Treatment with UFP-512 decreased the anxiogenic and depressant-like comportment associated with OA pain, and naltrindole reversed those effects. Anxiety- and depressive-like behaviors were assessed on day 29 following MIA or VEH injection in animals treated with 50 mg/kg of UFP-512, 3 mg/kg of naltrindole (NTD) or UFP-512 (50 mg/kg) plus naltrindole (3 mg/kg) in the EPM (**A**–**C**), TST (**D**) and FST (**E**). UFP-512 and naltrindole were given at 1 h and at 45 min before testing, respectively. The quantity of entrances into the open arms (**A**), percentage of time passed in the open arms (**B**) and the number of entrances into the closed arms (**C**) in the EPM and the immobility time (s) in the TST (**D**) and FST (**E**) are displayed. In each graph, symbols denote significant variations; * vs. VEH-VEH and $ vs. MIA-UFP-512 (*p* < 0.05; one-way ANOVA followed Tuckey test). Data are showed as mean ± SEM; *n* = 8 subjects.

To determine if UFP-512 could modulate the emotional-like states accompanying OA pain, we evaluated the effects of an elevated dose of this DOR agonist (50 mg/kg) on the anxiety- and depressive-like behaviors manifesting at 29 days after MIA-injection (Figure 3). In the EPM test, the significant differences between MIA- and VEH-injected mice in both the quantity of entrances into the open arms (Figure 3A) (*p* < 0.001 vs. VEH; one-way ANOVA) and in the proportion of time spent in them (Figure 3B) (*p* < 0.001 vs. VEH; one-way ANOVA) revealed anxious like-behaviors accompanying OA pain in mice.

The administration of UFP-512 normalized these anxiogenic-like responses, and its effects were reversed with the administration of naltrindole, showing the specificity of the anxiolytic effects of this drug. Naltrindole administered alone did not alter the anxiogenic-like behaviors associated with OA pain (Figure 3A,B). Moreover, the number of entries into the closed arms did not differ among groups (Figure 3C).

The TST and FST were used to evaluate the depressive-like behaviors related to OA pain, which were demonstrated by the increased immobility time of MIA-injected mice as compared with VEH-injected animals (Figure 3D,E) (*p* < 0.001 vs. their corresponding VEH; one-way ANOVA). In both tests, the administration of UFP-512 inhibited depressive-like comportment, and its effects were reversed by naltrindole. These findings revealed the anxiolytic and antidepressant actions of UFP-512 in mice with OA pain.

### 3.3. Effects of Treatment with UFP-512 on p-IKBα, SOD-1 and GSTM1 Levels in the DRG, AMG and HIP of Mice with OA Pain

**Figure 4:**
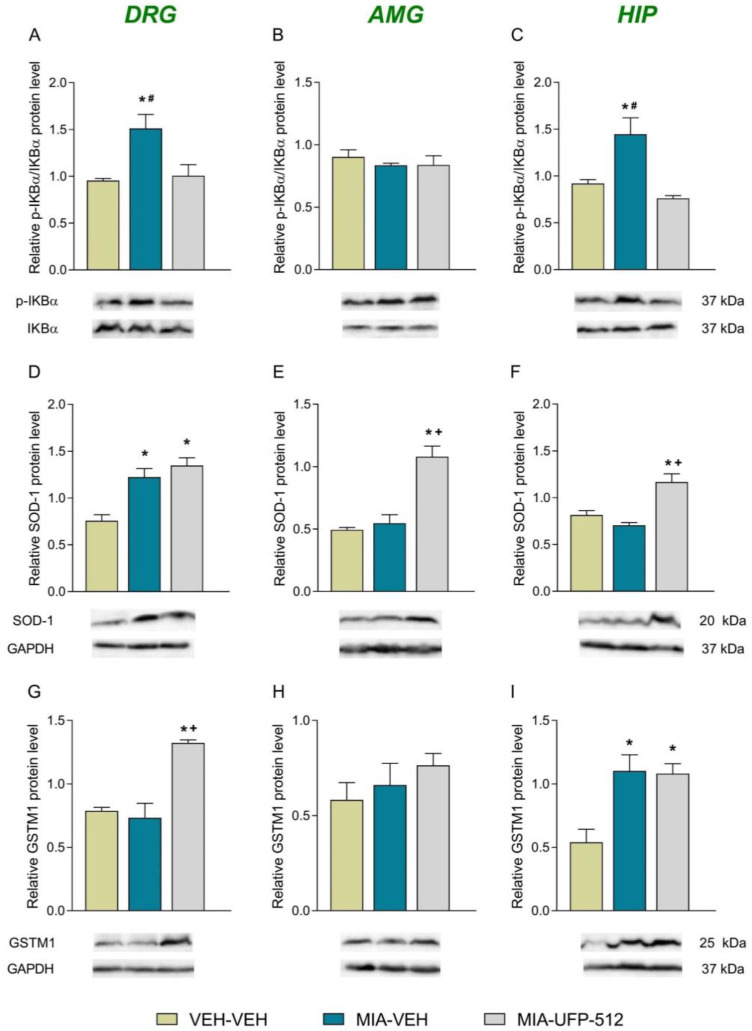
Effects of treatment with 50 mg/kg of UFP-512 given i.p. on the levels of p-IKBα, SOD-1 and GSTM1 in the DRG, AMG and HIP of animals with OA pain at 29 days of MIA injection. Animals treated with VEH-VEH were the controls. The expression of p-IKBα (**A**), SOD-1 (**D**), and GSTM1 (**G**) in the DRG; p-IKBα (**B**), SOD-1 (**E**), and GSTM1 (**H**) in the AMG; and p-IKBα (**C**), SOD-1 (**F**) and GSTM1 (**I**) in the HIP are represented. VEH-VEH-treated mice were utilized as controls. Representative blots for these proteins are displayed. All proteins are expressed relative to GAPDH levels excepting p-IKBα, which is expressed relative to total IKBα. In all pictures, symbols denote significant changes; * vs. VEH-VEH; + vs. MIA-VEH and # vs. MIA-UFP-512 (*p* < 0.05; one-way ANOVA plus Tukey test). Data are showed as mean values ± SEM; *n* = 3 samples/group.

Knee injection of MIA increased the expression of p-IKBα (*p* < 0.01; one-way ANOVA vs. VEH-VEH-treated mice; Figure 4A) and SOD-1 (*p* < 0.002; one-way ANOVA VEH-VEH-treated mice; Figure 4D) but not that of GSTM1 (Figure 4G) in the DRG. Moreover, while heightened levels of p-IKBα were normalized with UFP-512, those of SOD-1 remained high after UFP-512 treatment. This treatment also increased the expression of GSTM1 in the DRG (*p* < 0.001; one-way ANOVA vs. VEH-VEH- and MIA-VEH-treated mice; Figure 4G). The administration of UFP-512 further improved SOD-1 levels in the AMG (*p* < 0.01; one-way ANOVA vs. VEH-VEH- and MIA-VEH-injected animals; Figure 4E) but did not alter the expression of p-IKBα (Figure 4B) or GSTM1 (Figure 4H) in this brain area. Regarding HIP, our outcomes showed that while the enhanced levels of p-IKBα triggered by MIA were normalized via UPF-512 treatment (*p* < 0.008; one-way ANOVA; Figure 4C) and the up-regulation of GSTM1 provoked by MIA remained high after UFP-512 treatment (*p* < 0.001; one-way ANOVA; Figure 4I), the expression of SOD-1 increased in UFP-512-treated mice (*p* < 0.01; one-way ANOVA vs. VEH-VEH- and MIA-VEH-treated mice; Figure 4F).

### 3.4. Effects of Treatment with UFP-512 plus H_2_S Donors on Allodynia and GS Deficits Provoked by MIA

**Figure 5:**
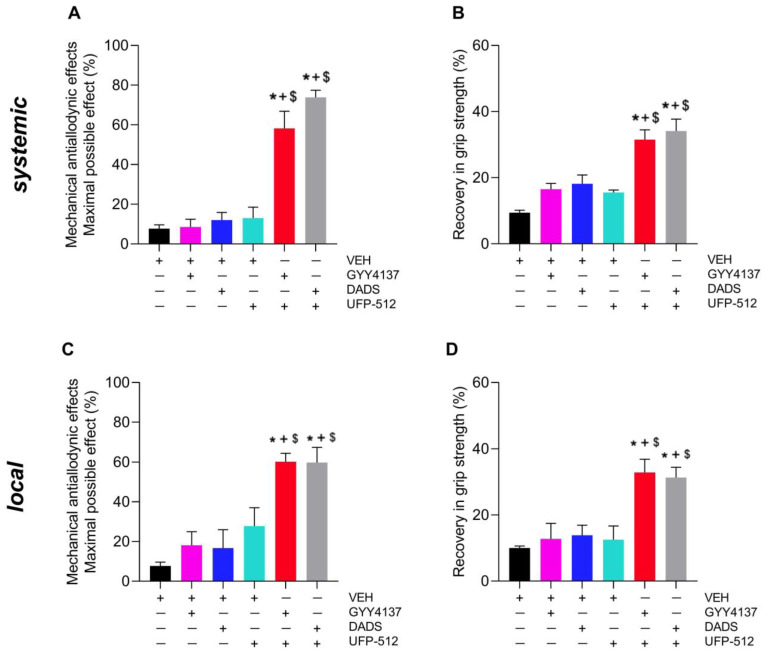
The impact of UFP-512 combined with two H_2_S donors on allodynia and GS deficits generated by MIA at 29 days after injection. The actions of the systemic (3 mg/kg) or local (250 µg) injection of UFP-512 administered alone and combined with GYY4137 (0.4 mg/kg) or DADS (3 mg/kg) given i.p. on allodynia (**A**,**C**) and GS deficits caused by MIA (**B**,**D**) are showed. All drugs were given at 1 h before testing. In each graph, symbols denote significant changes; * vs. VEH, + vs. GYY4137 or DADS and $ vs. UFP-512-injected mice (*p* < 0.05; one-way ANOVA plus Tuckey test). Results are showed as mean ± SEM; *n* = 6 subjects.

We evaluated the effects produced by the systemic (3 mg/kg) or local (250 µg) administration of UFP-512 alone and combined with subanalgesic doses of GYY4137 (0.4 mg/kg) or DADS (3 mg/kg) given i.p. on allodynia (Figure 5A,C) and GS deficits (Figure 5B,D) caused by MIA.

Data showed that the systemic administration of GYY4137 or DADS significantly heightened the anti-allodynic actions of UFP-512 given i.p. (*p* < 0.001, one-way ANOVA; Figure 5A) or i.a. (*p* < 0.001, one-way ANOVA; Figure 5C) as compared with the effects produced by VEH, UFP-512, GYY4137 or DADS administered separately.

In addition, the systemic (Figure 5B) and local (Figure 5D) administration of UFP-512 combined with GYY4137 or DADS given i.p. produced a greater recovery of GS than that produced by VEH, UFP-512, GYY4137 or DADS administered independently (*p* < 0.001, one-way ANOVA).

### 3.5. Impact of H_2_S on Levels of DORs in the DRG, AMG and HIP of MIA-Injected Mice

**Figure 6:**
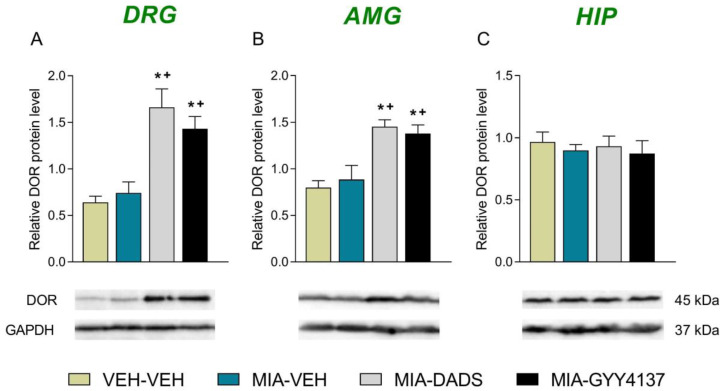
Effect of H_2_S on the levels of DORs in the DRG, AMG and HIP of MIA-injected mice. Treatment with 15 mg/kg of DADS and 6 mg/kg of GYY4137 both increased the expression of DORs in the DRG (**A**) and AMG (**B**) of animals with OA pain at 29 days after induction. No alterations in DOR expression were identified in the HIP (**C**). VEH-injected animals receiving VEH were the controls. In all pictures, symbols denote significant changes; * vs. VEH-VEH and + vs. MIA-VEH (*p* < 0.05; one-way ANOVA plus Tukey test). The results are showed as mean values ± SEM; *n* = 3 samples/group.

Our results showed that although no effects of OA on the expression of DORs in the DRG (Figure 6A), AMG (Figure 6B) or HIP (Figure 6C) were detected, the administration of DADS and GYY4137 significantly improved their expression in the DRG (*p* < 0.001, one-way ANOVA; Figure 6A) and AMG (*p* < 0.001, one-way ANOVA; Figure 6B) as compared with VEH-VEH- and MIA-VEH-treated animals.

## 4. Discussion

Knee OA is a chronic disorder that affects the joints and causes pain and notable incapacity problems. Although several pharmacological approaches are indicated to relieve some pain symptoms, there is not a satisfactory treatment for OA pain. In addition, the current therapies, besides not being completely effective, have many side effects. Moreover, OA pain is known to be associated with several functional and emotional impairments in patients [39]. Therefore, it is indispensable to find a global and efficient treatment for OA pain.

This study demonstrated that the systemic and local administration of a DOR agonist, UFP-512, inhibited the allodynia and GS deficits occasioned by OA in a dose-dependent manner. UFP-512 also inhibited the anxiodepressive-like behaviors related to OA pain, and all these effects were reversed with the administration of naltrindole, thus proving the participation of DORs in UFP-512 actions. Our findings further demonstrated that the co-treatment of low doses of UFP-512 with H_2_S donors heightens anti-allodynic effects and the recovery of the loss of GS produced by this DOR agonist. Furthermore, UFP-512 regulated the inflammatory and oxidative reactions generated by OA, and both DADS and GYY4137 enhanced DOR expression in the CNS and/or PNS.

The effectivity of DORs in inhibiting inflammatory and neuropathic pain have been well demonstrated [12,40], but its actions in OA pain are less well known. In this study, we demonstrated the anti-allodynic effects and the recovery of GS deficits produced by UFP-512 in animals with knee OA. Our results are in accordance with the painkilling actions of this and other DOR agonists (DPDPE or SNC-80) in rodents with inflammatory, cancer and neuropathic pain or with migraine [8,20] and further demonstrated the effectiveness of UFP-512 in inhibiting OA pain. The specificity of the local and systemic actions of UFP-512 in OA pain modulation has been demonstrated via its reversal with naltrindole. These data confirm the greater sensitivity of DOR knockout mice for developing neuropathy [41] and the non-effects of SNC-80 in DOR knockout mice under inflammatory pain conditions [18].

It is well recognized that one important problem for patients suffering OA is the accompanying functional disabilities and therapy. Thus, it is important to evaluate the effectiveness of a drug on the physical dysfunctions accompanying OA. Our data demonstrated a partial recovery of the loss of GS caused by MIA in animals treated with UFP-512 given i.p. or i.a. This is in accordance with the incomplete retrieval of GS produced by morphine (a MOR agonist) and other analgesics, NSAIDs or acetaminophen in rats with CFA-provoked GS loss [42], but in contrast with the complete reversion of the GS deficits produced by two H_2_S donors in MIA-injected mice [34]. Our data also demonstrated that the anti-allodynic effects and the recovery of GS resulting from the systemic or local injection of high doses of UFP-512 were reversed by naltrindole given i.p. or i.a., validating the specificity of this agonist interacting with central and peripheral DORs.

In addition, we assessed the co-administration of UFP-512 with low doses of DADS or GYY4137, two H_2_S donors, to see if they could augment the efficiency of this DOR agonist in inhibiting allodynia and GS deficits. Interestingly, the co-treatment with both H_2_S donors significantly improved anti-allodynic actions and the recovery of GS deficits produced by UFP-512 when systemically or locally administered. That is, the co-administration of UFP-512 with DADS or GYY4137 displayed greater anti-allodynic effects and a better recuperation of GS than did the same dose of this DOR agonist given separately. These data also corresponded with the strengthened anti-allodynic actions produced by UFP-512 and another DOR agonist combined with slow-releasing H_2_S donors during inflammatory and neuropathic pain [28,29]. The present study demonstrated a positive interaction between H_2_S and DORs in modulating the tactile allodynia and mechanical dysfunctions occasioned by OA in mice and thus allowed for the use of low doses of UFP-512 combined with DADS or GYY4137 for treating OA pain.

Our findings further revealed that both H_2_S donors improved the protein levels of DORs in the DRG and AMG, thus corroborating the relationship between this gas and the DOR system during chronic pain. Our data agree with the capacity of H_2_S to activate the expression of DORs in the paw and DRG of animals with peripheral inflammation or nerve injury-provoked inflammatory or neuropathic pain [28,29]. Our results suggest that the increased levels of DORs induced by DADS and GYY4137 in the DRG and AMG of mice with OA pain may play a significant role on the enhancement of anti-allodynic actions and/or in the recovery of GS produced by UFP-512 in mice co-treated with H_2_S donors. In addition, the limited adverse effects of DOR agonists suggest that the local and systemic administration of UFP-512 alone or in combination with H_2_S donors may be advantageous for the management of OA pain. The absence of alterations in the DOR protein levels in the HIP of MIA-injected mice treated with DADS or GYY4137 was in line with the absence of alterations in the levels of this receptor in the medial septum of nerve-injured animals treated with these H_2_S donors [28], suggesting that these brain regions are not fully implicated in the interaction between the DOR and H_2_S systems in chronic pain.

Earlier experiments further demonstrated the significance of the endogenous opioid system in the anti-allodynic actions of H_2_S donors during neuropathic and visceral pain [28,43]. These findings suggested that the favorable interaction between DOR agonists and the H_2_S system in palliating chronic pain might be a result not solely of the heightening of DOR expression resulting from H_2_S, but also of the activation of the endogenous opioid system induced by this gaseous neurotransmitter. Therefore, it is conceivable to propose that the activation of the endogenous opioid system stimulated by DADS or GYY4137 may also contribute to the enhancement of the anti-allodynic actions and the recovery of GS produced by UFP-512 when combined with these H_2_S donors in mice suffering OA pain.

The antidepressant and anxiolytic actions of DORs have been described in animals without pain and in several preclinical models of depressive- and anxiety-like states [44,45]. Therefore, DOR deficient mice exhibit anxiogenic- and depressive-like comportment [10], emphasizing the function of this receptor in reducing mood disorders. Nevertheless, the role of DORs in controlling the emotional disorders concurrent with OA have not been totally investigated. This work demonstrated that UFP-512 inhibits anxiogenic and depressive-like behaviors observed in MIA-injected mice and that these effects were reversed with naltrindole, demonstrating that the UFP-512 actions were mediated by activating DORs. That is, the normalization of the low visits and the few time that animals with OA pain treated with UFP-512 passed into the open arms of a maze were reversed by naltrindole. In our experiments, VEH-VEH- and MIA-VEH-treated mice exhibited a similar number of entries into the closed arms in the EPM, suggesting that MIA did not alter the locomotor activity of these animals. In contrast, other authors demonstrated a negative effect on the locomotion induced by MIA, which is age-dependent, showing that between 3 and 14 days after osteoarthritis induction, locomotion was more affected in old mice than in adult mice [46]. The possible divergences in the effects of MIA on locomotor activity between our experiments and those performed by Amodeo et al. (2022) [46] might be related to the age of the animals (6–8 weeks vs. 20 months) as well as to the time in which locomotor activity was evaluated (29 days vs. 14 days after MIA injection). Therefore, the non-changes in the number of visits of mice treated with UFP-512 to the closed arms in the EPM support the idea that the anxiolytic and antidepressant effects caused by this DOR agonist in mice with OA pain are independent of the possible effects of MIA on locomotor activity [47].

UFP-512 also stabilized the enhanced inactivity of MIA-injected mice in the TST and FST, as an indication of the antidepressant activity of this DOR agonist in animals with OA pain. In accordance with our findings, the antidepressant capacity of SNC80 has been exhibited by rats with inflammatory pain [48] and that of UFP-512 in mice with neuropathy [20]. Therefore, our data showed that UFP-512 likewise improves the allodynia and GS deficits provoked by OA and further alleviates the mood disorders related to OA pain. Considering the low side effects of DORs [44,45,49,50], the use of DOR agonists might be very beneficial for treating the anxiodepressive-like responses accompanying OA pain in a safe manner, avoiding the use of anxiolytics such as benzodiazepines and/or tricyclic antidepressants, which can have high adverse effects [2].

NF-κB transcription factor participates in several inflammatory illnesses. Thus, the activation of NF-κB in response to nerve damage or tissue inflammation stimulates the inflammatory responses that provoke neuropathic or OA pain [51,52,53]. Moreover, the deletion of the NF-κB gene reduced mechanical allodynia triggered by sciatic nerve injury by blocking the increased expression of several pro-inflammatory mediators in the spinal cord [54]. Other study further demonstrated that the increased spinal cord levels of NF-κB provoked by MIA were reduced with the administration of an NF-κB inhibitor, suggesting that the up-regulation of this inflammatory factor contributes to the development of OA pain caused by MIA [55]. In agreement with these results, increased levels of p-IKBα, an activator of NF-κB, were demonstrated in the DRG of MIA-injected mice; these data are reinforced by the up-regulation of pIKB-α and NF-κB detected in the joint tissues of rats with arthritis [56].

Other studies also indicate that the emotional disorders associated with chronic pain are related to inflammatory responses in some brain areas, such as the HIP, which plays a crucial role in controlling affective disorders [57]. Indeed, the up-regulation of p-IKBα observed in the HIP of MIA-injected mice might be related to the anxiodepressive-like behaviors observed in these animals, as occurs in arthritic rats [58]. Furthermore, the administration of UFP-512 reversed the up-regulation of p-IKBα in the DRG and HIP. Therefore, considering the notable role played by the p-IKBα-NF-kB pathway in the development of chronic pain and mood disorders, our data suggest that the normalization of the overexpression of p-IKBα in the DRG and HIP produced by UFP-512 might be involved in the inhibition of allodynia as well as of the emotional and/or mechanical deficits produced by this DOR agonist. In accordance with this suggestion, other treatments such as berberine and dimethyl fumarate also relieve gouty arthritis, neuropathy or post-operative pain by blocking the activation of p-IKBα-NF-kB pathway [59,60,61].

Oxidative stress and inflammation are closely linked, and both are significant contributors to the onset of joint pain [62]. Different works have indicated that inflammation increases the production of ROS which can stimulate the output of pro-inflammatory mediators [63,64]. In addition, other studies have showed that the activation of the Nrf2 signaling pathway diminished inflammatory pain [65], and UFP-512 activates the Nrf2 signaling pathway to exert its cytoprotective effects against hypoxic injury in cell cultures [32]. Then, we evaluated the expression of two antioxidant enzymes activated by Nrf2 transcription factor [31], SOD-1 and GSTM1 in the PNS and CNS of MIA-injected animals treated with UFP-512. Our results showed that the increased expression of SOD-1 and GSTM1 triggered by MIA in the DRG and HIP remained higher after UFP-512 treatment. Moreover, this DOR agonist was also able to potentiate the expression of GSTM1 in the DRG and that of SOD-1 in the AMG and HIP of animals with OA pain. These results coincide with the effects produced by other treatments, such as salvianolic acid B and decursin, which inhibit OA or inflammatory pain by activating the endogenous antioxidant signaling pathway [56,66]. Therefore, the capacity of UFP-512 to activate the peripheral and central antioxidant system in animals with OA pain might be implicated in its ability to inhibit the allodynia, the GS deficits and the anxiodepressive-like behaviors associated with OA. In conformity with this suggestion, UFP-512 also inhibited the depressive-like behaviors accompanying neuropathic pain by stimulating the endogenic antioxidant system [20].

## 5. Conclusions

In summary, our results revealed that the systemic and local administration of UFP-512 dose-dependently inhibited the allodynia and the loss of GS caused by MIA, effects which were potentiated by two H_2_S donors and reversed by naltrindole. UFP-512 also inhibited anxiodepressive-like behaviors, normalized the overexpression of p-IKBα in the DRG and HIP, and activated the expression of SOD-1 and GSTM1 in the DRG, HIP and/or AMG. Moreover, the enhanced expression of DORs triggered by H_2_S in the DRG and AMG might support the improved analgesic actions of UFP-512 co-administered with DADS or GYY4137. This study proposes the use of DOR agonists alone or combined with H_2_S donors as a new treatment for OA pain.

## Data Availability

Data is contained within the article.

## References

[1] Blanco F.J., Valdes A.M., Rego-Pérez I. (2018). Mitochondrial DNA variation and the pathogenesis of osteoarthritis phenotypes. Nat. Rev. Rheumatol..

[2] Bortoluzzi A., Furini F., Scirè C.A. (2018). Osteoarthritis and its management—Epidemiology, nutritional aspects and environmental factors. Autoimmun. Rev..

[3] Bannuru R.R., Osani M.C., Vaysbrot E.E., Arden N.K., Bennell K., Bierma-Zeinstra S.M.A., Kraus V.B., Lohmander L.S., Abbott J.H., Bhandari M. (2019). OARSI guidelines for the non-surgical management of knee, hip, and polyarticular osteoarthritis. Osteoarthr. Cartil..

[4] Jacobs C.A., Mace R.A., Greenberg J., Popok P.J., Reichman M., Lattermann C., Burris J.L., Macklin E.A., Vranceanu A.M. (2021). Development of a mind body program for obese knee osteoarthritis patients with comorbid depression. Contemp. Clin. Trials Commun..

[5] Yu H., Huang T., Lu W.W., Tong L., Chen D. (2022). Osteoarthritis Pain. Int. J. Mol. Sci..

[6] Cahill C.M., Taylor A.M., Cook C., Ong E., Morón J.A., Evans C.J. (2014). Does the kappa opioid receptor system contribute to pain aversion?. Front. Pharmacol..

[7] Bodnar R.J. (2022). Endogenous opiates and behavior: 2020. Peptides.

[8] Berthiaume S., Abdallah K., Blais V., Gendron L. (2020). Alleviating pain with delta opioid receptor agonists: Evidence from experimental models. J. Neural Transm..

[9] Tsukahara-Ohsumi Y., Tsuji F., Niwa M., Nakamura M., Mizutani K., Inagaki N., Sasano M., Aono H. (2010). SA14867, a newly synthesized kappa-opioid receptor agonist with antinociceptive and antipruritic effects. Eur. J. Pharmacol..

[10] Filliol D., Ghozland S., Chluba J., Martin M., Matthes H.W., Simonin F., Befort K., Gavériaux-Ruff C., Dierich A., LeMeur M. (2000). Mice deficient for delta- and mu-opioid receptors exhibit opposing alterations of emotional responses. Nat. Genet..

[11] Ragnauth A., Schuller A., Morgan M., Chan J., Ogawa S., Pintar J., Bodnar R.J., Pfaff D.W. (2001). Female preproenkephalin-knockout mice display altered emotional responses. Proc. Natl. Acad. Sci. USA.

[12] Browne C.A., Lucki I. (2019). Targeting opioid dysregulation in depression for the development of novel therapeutics. Pharmacol. Ther..

[13] Olson K.M., Hillhouse T.M., Burgess G.E., West J.L., Hallahan J.E., Dripps I.J., Ladetto A.G., Rice K.C., Jutkiewicz E.M., Traynor J.R. (2023). Delta Opioid Receptor-Mediated Antidepressant-Like Effects of Diprenorphine in Mice. J. Pharmacol. Exp. Ther..

[14] Al-Hasani R., Bruchas M.R. (2011). Molecular mechanisms of opioid receptor-dependent signaling and behavior. Anesthesiology.

[15] Gavériaux-Ruff C., Kieffer B.L. (2011). Delta opioid receptor analgesia: Recent contributions from pharmacology and molecular approaches. Behav. Pharmacol..

[16] Cahill C.M., Holdridge S.V., Morinville A. (2007). Trafficking of delta-opioid receptors and other G-protein-coupled receptors: Implications for pain and analgesia. Trends Pharmacol. Sci..

[17] Jimenez-Vargas N.N., Gong J., Wisdom M.J., Jensen D.D., Latorre R., Hegron A., Teng S., DiCello J.J., Rajasekhar P., Veldhuis N.A. (2020). Endosomal signaling of delta opioid receptors is an endogenous mechanism and therapeutic target for relief from inflammatory pain. Proc. Natl. Acad. Sci. USA.

[18] Gavériaux-Ruff C., Karchewski L.A., Hever X., Matifas A., Kieffer B.L. (2008). Inflammatory pain is enhanced in delta opioid receptor-knockout mice. Eur. J. Neurosci..

[19] Pellissier L.P., Pujol C.N., Becker J.A.J., Le Merrer J. (2018). Delta Opioid Receptors: Learning and Motivation. Handb. Exp. Pharmacol..

[20] Polo S., Díaz A.F., Gallardo N., Leánez S., Balboni G., Pol O. (2019). Treatment with the Delta Opioid Agonist UFP-512 Alleviates Chronic Inflammatory and Neuropathic Pain: Mechanisms Implicated. Front. Pharmacol..

[21] Choi M.C., Jo J., Park J., Kang H.K., Park Y. (2019). NF-κB Signaling Pathways in Osteoarthritic Cartilage Destruction. Cells.

[22] Kabe Y., Ando K., Hirao S., Yoshida M., Handa H. (2005). Redox regulation of NF-kappaB activation: Distinct redox regulation between the cytoplasm and the nucleus. Antioxid. Redox Signal..

[23] Yang H., Wang Z., Wang L., Li Y., Guo J., Yang X., Zhao J., Rong K., Zhang P., Ye B. (2022). Scutellarin ameliorates osteoarthritis by protecting chondrocytes and subchondral bone microstructure by inactivating NF-κB/MAPK signal transduction. Biomed. Pharmacother..

[24] Feng J.H., Jung J.S., Hwang S.H., Lee S.K., Lee S.Y., Kwak Y.G., Kim D.H., Song C.Y., Kim M.J., Suh H.W. (2022). The mixture of Agrimonia pilosa Ledeb. and Salvia miltiorrhiza Bunge. extract produces analgesic and anti-inflammatory effects in a collagen-induced arthritis mouse model. Anim. Cells Syst..

[25] Lucarini E., Micheli L., Martelli A., Testai L., Calderone V., Ghelardini C., Di Cesare Mannelli L. (2018). Efficacy of isothiocyanate-based compounds on different forms of persistent pain. J. Pain Res..

[26] Guo J., Li G., Yang L. (2020). Role of H2S in pain: Growing evidences of mystification. Eur. J. Pharmacol..

[27] Shayea A.M.F., Mousa A.M.A., Renno W.M., Nadar M.S., Qabazard B., Yousif M.H.M. (2020). Chronic Treatment with Hydrogen Sulfide Donor GYY4137 Mitigates Microglial and Astrocyte Activation in the Spinal Cord of Streptozotocin-Induced Diabetic Rats. J. Neuropathol. Exp. Neurol..

[28] Bai X., Batallé G., Balboni G., Pol O. (2022). Hydrogen Sulfide Increases the Analgesic Effects of µ- and δ-Opioid Receptors during Neuropathic Pain: Pathways Implicated. Antioxidants.

[29] Porta A., Rodríguez L., Bai X., Batallé G., Roch G., Pouso-Vázquez E., Balboni G., Pol O. (2021). Hydrogen Sulfide Inhibits Inflammatory Pain and Enhances the Analgesic Properties of Delta Opioid Receptors. Antioxidants.

[30] Wang Z., Liang M., Li H., Cai L., He H., Wu Q., Yang L. (2019). l-Methionine activates Nrf2-ARE pathway to induce endogenous antioxidant activity for depressing ROS-derived oxidative stress in growing rats. J. Sci. Food Agric..

[31] Basu P., Averitt D.L., Maier C., Basu A. (2022). The Effects of Nuclear Factor Erythroid 2 (NFE2)-Related Factor 2 (Nrf2) Activation in Preclinical Models of Peripheral Neuropathic Pain. Antioxidants.

[32] Cao S., Chao D., Zhou H., Balboni G., Xia Y. (2015). A novel mechanism for cytoprotection against hypoxic injury: δ-opioid receptor-mediated increase in Nrf2 translocation. Br. J. Pharmacol..

[33] Balboni G., Salvadori S., Guerrini R., Negri L., Giannini E., Jinsmaa Y., Bryant S.D., Lazarus L.H. (2002). Potent delta-opioid receptor agonists containing the Dmt-Tic pharmacophore. J. Med. Chem..

[34] Batallé G., Bai X., Pol O. (2022). The Interaction between Carbon Monoxide and Hydrogen Sulfide during Chronic Joint Pain in Young Female Mice. Antioxidants.

[35] Chaplan S.R., Bach F.W., Pogrel J.W., Chung J.M., Yaksh T.L. (1994). Quantitative assessment of tactile allodynia in the rat paw. J. Neurosci. Methods.

[36] Walf A.A., Frye C.A. (2007). The use of the elevated plus maze as an assay of anxiety-related behavior in rodents. Nat. Protoc..

[37] Steru L., Chermat R., Thierry B., Simon P. (1985). The tail suspension test: A new method for screening antidepressants in mice. Psychopharmacology.

[38] Porsolt R.D., Brossard G., Hautbois C., Roux S. (2001). Rodent models of depression: Forced swimming and tail suspension behavioral despair tests in rats and mice. Curr. Protoc. Neurosci..

[39] Stubbs B., Vancampfort D., Veronese N., Thompson T., Fornaro M., Schofield P., Solmi M., Mugisha J., Carvalho A.F., Koyanagi A. (2017). Depression and pain: Primary data and meta-analysis among 237,952 people across 47 low- and middle-income countries. Psychol. Med..

[40] Abdallah K., Gendron L. (2018). The Delta Opioid Receptor in Pain Control. Handb. Exp. Pharmacol..

[41] Nadal X., Baños J.E., Kieffer B.L., Maldonado R. (2006). Neuropathic pain is enhanced in delta-opioid receptor knockout mice. Eur. J. Neurosci..

[42] Montilla-García Á., Tejada M.Á., Ruiz-Cantero M.C., Bravo-Caparrós I., Yeste S., Zamanillo D., Cobos E.J. (2019). Modulation by Sigma-1 Receptor of Morphine Analgesia and Tolerance: Nociceptive Pain, Tactile Allodynia and Grip Strength Deficits During Joint Inflammation. Front. Pharmacol..

[43] Distrutti E., Cipriani S., Renga B., Mencarelli A., Migliorati M., Cianetti S., Fiorucci S. (2010). Hydrogen sulphide induces micro opioid receptor-dependent analgesia in a rodent model of visceral pain. Mol. Pain.

[44] Pradhan A.A., Befort K., Nozaki C., Gavériaux-Ruff C., Kieffer B.L. (2011). The delta opioid receptor: An evolving target for the treatment of brain disorders. Trends Pharmacol. Sci..

[45] Dripps I.J., Jutkiewicz E.M. (2018). Delta Opioid Receptors and Modulation of Mood and Emotion. Handb. Exp. Pharmacol..

[46] Amodeo G., Franchi S., Galimberti G., Comi L., D’Agnelli S., Baciarello M., Bignami E.G., Sacerdote P. (2022). Osteoarthritis Pain in Old Mice Aggravates Neuroinflammation and Frailty: The Positive Effect of Morphine Treatment. Biomedicines.

[47] Vergura R., Balboni G., Spagnolo B., Gavioli E., Lambert D.G., McDonald J., Trapella C., Lazarus L.H., Regoli D., Guerrini R. (2008). Anxiolytic- and antidepressant-like activities of H-Dmt-Tic- NH-CH(CH2-COOH)-Bid (UFP-512), a novel selective delta opioid receptor agonist. Peptides.

[48] Chen C.M., Ding H., Mabry K.M., Ko M.C. (2022). Enhanced antidepressant-like effects of a delta opioid receptor agonist, SNC80, in rats under inflammatory pain. Pharmacol. Biochem. Behav..

[49] Pradhan A.A., Smith M.L., Zyuzin J., Charles A. (2014). δ-Opioid receptor agonists inhibit migraine-related hyperalgesia, aversive state and cortical spreading depression in mice. Br. J. Pharmacol..

[50] Gallantine E.L., Meert T.F. (2005). A comparison of the antinociceptive and adverse effects of the mu-opioid agonist morphine and the delta-opioid agonist SNC80. Basic Clin. Pharmacol. Toxicol..

[51] Fu E.S., Zhang Y.P., Sagen J., Candiotti K.A., Morton P.D., Liebl D.J., Bethea J.R., Brambilla R. (2010). Transgenic inhibition of glial NF-kappa B reduces pain behavior and inflammation after peripheral nerve injury. Pain.

[52] Luo J.G., Zhao X.L., Xu W.C., Zhao X.J., Wang J.N., Lin X.W., Sun T., Fu Z.J. (2014). Activation of spinal NF-κB/p65 contributes to peripheral inflammation and hyperalgesia in rat adjuvant-induced arthritis. Arthritis Rheumatol..

[53] Lee K.M., Kang B.S., Lee H.L., Son S.J., Hwang S.H., Kim D.S., Park J.S., Cho H.J. (2004). Spinal NF-kB activation induces COX-2 upregulation and contributes to inflammatory pain hypersensitivity. Eur. J. Neurosci..

[54] Sun T., Luo J., Jia M., Li H., Li K., Fu Z. (2012). Small interfering RNA-mediated knockdown of NF-κBp65 attenuates neuropathic pain following peripheral nerve injury in rats. Eur. J. Pharmacol..

[55] Li Y., Yang Y., Guo J., Guo X., Feng Z., Zhao X. (2020). Spinal NF-kB upregulation contributes to hyperalgesia in a rat model of advanced osteoarthritis. Mol. Pain.

[56] Xia Z.B., Yuan Y.J., Zhang Q.H., Li H., Dai J.L., Min J.K. (2018). Salvianolic Acid B Suppresses Inflammatory Mediator Levels by Downregulating NF-κB in a Rat Model of Rheumatoid Arthritis. Med. Sci. Monit..

[57] Levy M.J.F., Boulle F., Steinbusch H.W., van den Hove D.L.A., Kenis G., Lanfumey L. (2018). Neurotrophic factors and neuroplasticity pathways in the pathophysiology and treatment of depression. Psychopharmacology.

[58] Zhu L., Chen T., Chang X., Zhou R., Luo F., Liu J., Zhang K., Wang Y., Yang Y., Long H. (2016). Salidroside ameliorates arthritis-induced brain cognition deficits by regulating Rho/ROCK/NF-κB pathway. Neuropharmacology.

[59] Hashemzaei M., Rezaee R. (2021). A review on pain-relieving activity of berberine. Phytother. Res..

[60] Casili G., Lanza M., Filippone A., Cucinotta L., Paterniti I., Repici A., Capra A.P., Cuzzocrea S., Esposito E., Campolo M. (2022). Dimethyl Fumarate (DMF) Alleviated Post-Operative (PO) Pain through the N-Methyl-d-Aspartate (NMDA) Receptors. Antioxidants.

[61] Cao Y., Hu Y., Jin X.F., Liu Y., Zou J.M. (2023). Dimethyl fumarate attenuates MSU-induced gouty arthritis by inhibiting NLRP3 inflammasome activation and oxidative stress. Eur. Rev. Med. Pharmacol. Sci..

[62] Hitchon C.A., El-Gabalawy H.S. (2004). Oxidation in rheumatoid arthritis. Arthritis Res. Ther..

[63] Choy E. (2012). Understanding the dynamics: Pathways involved in the pathogenesis of rheumatoid arthritis. Rheumatology.

[64] Arii K., Kumon Y., Sugahara K., Nakatani K., Ikeda Y., Suehiro T., Hashimoto K. (2008). Edaravone inhibits collagen-induced arthritis possibly through suppression of nuclear factor-kappa B. Mol. Immunol..

[65] Luan Y., Luo Y., Deng M. (2023). New advances in Nrf2-mediated analgesic drugs. Phytomedicine.

[66] He L., Pan Y., Yu J., Wang B., Dai G., Ying X. (2021). Decursin alleviates the aggravation of OA via inhibiting PI3K-Akt and NF-kB signal pathway. Int. Immunopharmacol..

